# Music in Waiting Rooms: A Literature Review

**DOI:** 10.1177/19375867211067542

**Published:** 2021-12-27

**Authors:** James C.-Y. Lai, Noel Amaladoss

**Affiliations:** 1 Michael G. DeGroote School of Medicine, McMaster University, Hamilton, Ontario, Canada; 2 Department of Psychiatry and Behavioural Neurosciences, McMaster University, Hamilton, Ontario, Canada

**Keywords:** emergency department, environment of care, hospital, literature review, mental health, music therapy

## Abstract

**Objective:**

We aim to review existing literature on the effects of background music in waiting rooms on patients. Furthermore, we examine existing neurobiological research for potential mechanisms by which music may affect patients.

**Background:**

Music has been studied in healthcare in various forms, from formal interventions such as music therapy to passive listening as therapy. However, music is also present in the healthcare environment in the form of background music in waiting rooms. There has been interest in whether background music in such a setting may have beneficial effects on patient anxiety in order to potentially inform healthcare workers whether and what type of music may be suitable for waiting rooms.

**Methods:**

We reviewed existing literature on music in healthcare waiting rooms and the neurobiological mechanisms by which music affects anxiety.

**Results:**

We located several small studies performed in a range of settings, including physician office waiting rooms and preoperative waiting areas. The studies generally reported that most patients viewed music in these areas positively; some, but not all, studies showed positive effects on patient anxiety. A variety of theories by which music may impact patient anxiety was noted.

**Conclusions:**

We conclude that there exists some evidence to support an anxiety-reducing effect of background music on patients, though studies vary widely in methodology and music selection. A small amount of neurobiological research into the pertinent mechanisms has been conducted, but further research will be required to elucidate the exact mechanisms by which this intervention may reduce anxiety.

Music is omnipresent in society. From concerts to social gatherings, in shopping establishments and workplaces, people listen to a variety of music, in a multitude of settings, and for many reasons. One particular application of music that has been the subject of some study has been the effects of background music. This is particularly well-studied in commercial shopping settings (Hargreaves & Krause, 2016), where it has been found that classical music can create an impression of a more sophisticated atmosphere, which in turn can increase listener spending, and that louder and faster music leads to consumers acting more quickly and spending less time in a venue. Personal use of music in the workplace has also been studied; for example, it has been found that music that workers either strongly like or dislike decreases their attention (Huang & Shih, 2011) and that the presence of lyrics negatively affects attention (Shih et al., 2012). However, background music is also present in a variety of medical settings, and these have not been as well-studied.

Music can be found in a number of medical settings, both as an intervention and as background music. Music has been used in mindfulness-based interventions (Donovan et al., 2019; Duchemin et al., 2015; van Emmerik et al., 2018), generally combined with or compared to formal music therapy (Liu et al., 2019; Shallcross et al., 2018). Aside from mindfulness-based interventions, music has also been studied in the treatment of depression, both as self-guided listening for patients and in more active formats like music therapy (Leubner & Hinterberger, 2017). Meanwhile, background music is present in settings such as clinic waiting rooms and operating theaters (e.g., Shakir et al., 2018). Given music’s use for relaxation in other settings, the reduction of patient anxiety is one particular area of interest for the use of background music in such settings. This review will focus on the effects of background music specifically in the setting of waiting rooms, that is, music played in waiting room settings for passive listening, rather than the use of music as a component of an active therapeutic modality.

In this review, we aim to summarize the current state of research into background music in the setting of healthcare waiting rooms. We discuss neurobiology that may be relevant for understanding the effects of background music on listeners in these settings. Finally, we discuss the possible implications on whether and what type, if any, of background music is suited to waiting rooms as well as areas that may potentially be of interest for future research.

## Clinical Studies and Surveys

One setting in which background music is present in the medical world is in waiting rooms. A common reason cited for playing music in such a setting is to reduce anxiety among patients awaiting clinical encounters. Indeed, studies of the psychology of music in general find that one way in which people use music is for relaxation (Schäfer et al., 2013). However, whether this is actually a successful intervention in the waiting room setting has not been well-studied. Most current studies have been conducted on small samples.

In one study conducted in the waiting room of a plastic surgeon in Germany, a total of 117 patients were exposed to one of four conditions: background instrumental music containing nature sounds paired with ambient lavender scent, either alone or neither. This study concluded that either intervention alone decreased anxiety in patients in a statistically significant manner, but when combined, there was no longer a significant difference from being exposed to neither intervention. The authors theorized this may be explained by the combination of multiple stimuli leading to increased arousal in patients, rather than decreasing arousal as when presented individually (Fenko & Loock, 2014). Another study similar in design performed in a pediatric emergency waiting room found that classical music (“geared toward relaxation,” mostly with tempi ranging from 60 to 70 beats per minute) alone or with aromatherapy decreased anxiety scores of patients’ parents, whereas no difference was seen for aromatherapy alone. However, the study did not evaluate the effect on the children, that is, the patients themselves (Holm & Fitzmaurice, 2008). This result is consistent with a survey that indicated that Western classical music in hospital waiting rooms decreased visitors’ self-reported stress levels (Routhieaux & Tansik, 1997). Meanwhile, a survey in India conducted by Panda et al. (2015) on children’s preferences for dentist office waiting rooms included the finding that children wished to have music playing in waiting rooms. The type of music preferred varied: Instrumental music was preferred over songs with words by older children (9–11 vs. 6–8 years old) and boys. Another survey conducted in a primary care waiting room likewise found that a majority of patients expressed a preference for music in waiting rooms, with classical music being most preferred. However, contradictorily, free-form comments collected during the study were “overwhelmingly negative,” though the authors did not speculate as to the reason for this. This study also found no significant difference in health or anxiety between patients exposed to waiting room music and those who were not (Jones & Brittain, 2009). In a mental health waiting room, similar findings of improved satisfaction but no change in anxiety were reported with the use of a piece of Western classical music (Waldon & Thom, 2015). The use of live music was explored in a study in a university health clinic, which found that patients exposed to live Western popular vocal music, performed by a music therapy student with guitar self-accompaniment, reported greater satisfaction with their visit and would be more likely to recommend the clinic to others (Silverman et al., 2012).

Background music has also been studied for its potential to reduce anxiety in preoperative waiting room settings. In one survey of preoperative patients, a majority indicated a preference for listening to music while awaiting surgery, though the type of music was not specified in the survey (Hyde et al., 1998). In a clinical study with no controls, a majority of patients exposed to music in the preanesthetic waiting room reported satisfaction with the intervention (Verheecke & Troch, 1980). A controlled study has also demonstrated that music (patient-selected from a variety of classical, environmental, New Age, country, and easy listening music) in the preoperative period significantly lowered patients’ heart rate, which was used as a surrogate for anxiety (Augustin & Hains, 1996). More recently, a study conducted on a sample of 159 patients in a single university medical center found statistically significant changes in various vital signs indicative of decreased anxiety when exposed to background classical or New Age music in the immediate preoperative period (Kipnis et al., 2016). Most patients exposed to the music intervention also reported an opinion that the music decreased their anxiety (Kipnis et al., 2016). On the other hand, a study conducted in a single Hong Kong community hospital found that listening to a patient’s choice of Eastern- or Western-style easy listening or Chinese pop music in the waiting room prior to day procedures only produced statistically significant decreased self-reported anxiety scores, with no significant difference in physiological parameters between the intervention and control groups (Lee et al., 2004). When considered together in a meta-analysis, a statistically and clinically significant decrease in State Trait Anxiety Inventory (STAI) score was noted among patients listening to music in preoperative settings (Brandt et al., 2013). Passive music-listening in obstetrical patients awaiting amniocentesis has also been studied. When patients were exposed to their choice of one of light English-language vocal music, light instrumental music, Western classical music, or English-language vocal jazz, anxiety, as measured using serum cortisol and STAI, was significantly lower relative to that in participants who read magazines or waited without either intervention (Ventura et al., 2012).

Overall, these results suggest that patients generally view music in waiting rooms positively, though impacts on anxiety, which were quantified using varying objective and subjective measures, were inconsistent between studies. However, a meta-analysis of studies that used the STAI reported that music reduced scores by 5.1 ± 0.53; a change of 5 points is generally considered to be clinically significant (Biddiss et al., 2014).

## Neurobiology of Music’s Effect on Anxiety

Listening to music is known to affect neurological activity, which may help to elucidate the mechanism by which music impacts patients’ anxiety. By better understanding the neurobiological basis behind these effects, it may become clearer which elements of music are important to determining its efficacy as an intervention, which may in turn guide the design and music choice of future studies.

Höller et al. (2012) examined the electroencephalogram (EEG) activity of subjects listening to relaxing and activating music of their own choice, including pop, classical, soul, and rock music for relaxation (of which five had no lyrics), and rock, techno, ska, and classical music for activation (of which only one had no lyrics). They noted that EEG changes varied between subjects, making it difficult to generalize the EEG effects of listening to different types of music. They did, however, note that an increase in beta range activity, which is associated with increased attention, tended to occur in those listening to activating music (Höller et al., 2012). Another study found that alpha brain waves, associated with relaxation, most increased relative to beta brain waves in subjects listening to classical music when compared to a variety of other genres, and that this effect was greater for the music of Mozart (and Beethoven to a lesser extent) when compared to Indian classical music (Ramdinmawii & Mittal, 2017). However, statistical analyses of the data were not performed, so it is unclear if these differences were significant. It has also been noted that music can reduce activity in areas of the brain associated with anxiety (Schäfer et al., 2013). In a study reviewing literature that examined neural activity changes in patients with anxiety and without anxiety, it was noted that amygdala activity was influenced by music, with consonant music (music with pitch combinations generally felt to be pleasing, as opposed to dissonant music, which contains pitch combinations that create a sense of tension) decreasing amygdala activity. It has previously been found that amygdala activity can be related to anxiety in subjects. However, there are currently no radioligand studies specifically examining the effect of music on γ-aminobutyric acid in the amygdala, which is the neurotransmitter related to the anxiety response (Archie et al., 2013).

Based on these findings, it appears that music that patients self-select as relaxing, and perhaps Western classical music by Mozart and Beethoven, may be better suited for relaxing patients. It is further suggested that consonant harmonic structure may be an important element contributing to a particular music selection’s anxiolytic properties. Further studies will be needed to confirm these findings more rigorously.

While there is a paucity of direct neurobiological imaging investigating music’s effects on anxiety-related brain function, several potential mechanisms have been proposed. One of these is alluded to by Fenko and Loock (2014), who noted that one mechanism by which music is believed to alleviate anxiety is by distraction. Furthermore, reference is made to the optimum arousal theory, which suggests that music lowers an anxious listener’s arousal level. Indeed, music has been shown to decrease arousal (Pelletier, 2004). Another theory suggests that music acts by eliciting positive emotions, which sensitizes the lateral hypothalamus, increasing vagal tone and parasympathetic stimulation of the heart, resulting in the observed decrease in heart rate associated with decreased anxiety in study participants. The involvement of parasympathetic regulation was noted to be supported by observed increases in nitric oxide signaling when listening to familiar self-selected music, though the specific types of music subjects selected were not reported (Ribeiro et al., 2018). This offers further support that self-selected music may be advantageous for reducing anxiety.

## Discussion

Overall, studies suggest that music in waiting rooms is at least well-tolerated by patients and may even reduce their anxiety. However, these conclusions are limited by the studies’ small sample sizes and disparate methodologies. In particular, some studies used ambient music in the waiting room while others examined music played on headphones. Furthermore, the music played for patients varied between studies, though it often consisted of some form of Western classical music. Music selection in most studies was not done in a rigorous process; in many studies, the methods by which music was selected were not described, while a few studies described reasoning justifying their selection of music that met characteristics previously found to be considered relaxing. Criteria described in such studies included diatonic melody (lacking notes outside of the traditional diatonic scale), predictable dynamic changes, consistent rhythm, and lack of lyrics (Waldon & Thom, 2015). Music having a tempo of approximately 60 bpm, or below 72 bpm, has also been described as a criterion for being relaxing (Holm & Fiztmaurice, 2008; Waldon & Thom, 2015). It has been noted that faster tempi of 100–120 bpm lead to sympathetic nervous system stimulation, while those slower than the average heart rate, approximately 60 bpm, may induce suspense and thus also not be favorable for relaxation (Routhieaux & Tansik, 1997). One laboratory-based study has reported that the effect of slow music tempo may be independent of musical genre (Bernardi et al., 2006). Only one study described a process by which music was selected through a formalized methodology of having a separate pool of participants rate music for its relaxing properties, which found that instrumental music containing nature sounds was considered more pleasant and relaxing than Western classical music (Fenko & Loock, 2014). More rigorously conducted trials with more standardized methodology and larger sample sizes would help further elucidate the effects of different types of music in waiting rooms.

***Overall, studies suggest that music in waiting rooms is at least well-tolerated by patients and may even reduce their anxiety.***

Neurobiologically, various potential mechanisms by which music may reduce anxiety in listeners have been explored. These include changes in activity in the amygdala as well as in vagal and parasympathetic tone. These advancements in neurobiology have added on to prior understanding, which consisted of various theories, such as those suggesting that music exerts its anxiolytic effect by distracting listeners. Although current understanding of the neurobiology behind these effects is incomplete, it appears that music self-selected for relaxing properties may be more associated with brain activity associated with relaxation. Further advancements in neuroimaging studies in this area will undoubtedly continue to improve the understanding of the mechanisms by which music reduces anxiety in patients.

How might this inform clinicians? Ultimately, the evidence suggests that music in waiting rooms could benefit patients by reducing anxiety, with minimal negative outcomes. Thus, it appears reasonable to play music in waiting room settings for patients. Previous studies have demonstrated that music in these settings can be played on headphones or speakers without significant differences in outcome (Lee et al., 2012). However, the choice of music is less clear; while research suggests certain qualities of music are associated with being more relaxing such as a slow tempo and low pitch (Kipnis et al., 2016), few clinical studies have directly compared different types of music in waiting room settings for anxiety reduction. The only study reviewed in this work that made such a comparison found no statistically significant difference between classical and New Age music (Kipnis et al., 2016). Outside of the clinical setting, one study employing EEG analysis of alpha wave activity suggested that classical music, and especially that of Mozart, may have stronger effects on inducing relaxation. Thus, it appears that any relaxing music, with characteristics such as a slow tempo around 60 bpm, predictable dynamics and rhythm, and consonant melodic and harmonic structure, may be a good choice for the waiting room setting. However, Western classical music, as the most well-studied option, may be a particular genre to consider for this purpose, although this could also simply reflect a cultural bias in music selection in studies.

It has been reported that, when surveyed, patients express their preference for the ability to select their own music and to control its volume (Augustin & Hains, 1996; Jones & Brittain, 2009). This has been used as an argument for playing music via headphones and allowing patients to select the music played. On the other hand, Holm and Fitzmaurice (2008) report that music played ambiently for all patients and parents in an emergency department was also well-tolerated. Overall, clinicians hoping to employ music in their waiting rooms may need to consider these differing findings, as well as the potential logistical issues in their waiting rooms such as infection control, when deciding the best delivery method of music in their waiting rooms (Biddiss et al., 2014).

An understanding of music’s effects on anxiety may also be helpful in exploring potential interventions in disease states such as anxiety disorders. In generalized anxiety disorders, background music has been used as a relaxation technique (Wetherell, 1998). However, there appears to be relatively little literature directly examining the efficacy of this intervention; the majority of literature instead focuses on active music therapy (e.g., Goldbeck & Ellerkamp, 2012; Gutiérrez & Camarena, 2015). Given that background music has been shown to reduce anxiety in a general waiting room population, it may be of interest to specifically examine whether this benefit can also be applied to patients with anxiety disorders.

## Conclusion

In summary, background music in waiting rooms appears to be a potentially beneficial intervention for reducing patient anxiety that is generally well-tolerated. There is a lack of literature to guide music selection, but relaxing music, such as Western classical music, is best studied to have shown benefit. Further studies on the impacts of background music on anxiety may also have potential benefits for informing treatment of some psychiatric disorders. We recommend larger scale studies to confirm these results and provide guidance on the optimal choice of music to inform clinicians on how best to implement this in their clinical settings.

## Implications for Practice

- Healthcare practitioners may consider the use of music in waiting rooms to reduce anxiety in patients.
- Preferred characteristics of music used in waiting rooms include slower tempo, consonant harmonic characteristics, and predictable dynamics and rhythms.
- Western classical music is best studied for this use, and the music of Mozart may be particularly efficacious.
- Delivery via headphones versus ambient music may be equally efficacious: While headphones may allow individual patient choice, which has been shown to be preferred by patients, this needs to be weighed against factors such as availability of equipment.

## References

[bibr1-19375867211067542] Archie P. Bruera E. Cohen L. (2013). Music-based interventions in palliative cancer care: A review of quantitative studies and neurobiological literature. Supportive Care in Cancer, 21(9), 2609–2624. 10.1007/s00520-013-1841-4 23715815 PMC3728458

[bibr2-19375867211067542] Augustin P. Hains A. A. (1996). Effect of music on ambulatory surgery patients’ preoperative anxiety. AORN Journal, 63, 750–758. 10.1016/S0001-2092(06)63126-8 8660020

[bibr3-19375867211067542] Bernardi L. Porta C. Sleight P. (2006). Cardiovascular, cerebrovascular, and respiratory changes induced by different types of music in musicians and non-musicians: The importance of silence. Heart, 92(4), 445–452. 10.1136/hrt.2005.064600 16199412 PMC1860846

[bibr4-19375867211067542] Biddiss E. Knibbe T. J. McPherson A. (2014). The effectiveness of interventions aimed at reducing anxiety in health care waiting spaces: A systematic review of randomized and nonrandomized trials. Anesthesia & Analgesia, 119(2), 433–448. 10.1213/ane.0000000000000294 24942321

[bibr5-19375867211067542] Brandt J. Dileo C. Shim M. (2013). Music interventions for preoperative anxiety. Cochrane Database of Systematic Reviews, 6(6), CD006908. 10.1002/14651858.cd006908.pub2 PMC9758540 23740695

[bibr6-19375867211067542] Donovan E. Martin S. R. Seidman L. C. Zeltzer L. K. Cousineau T. M. Payne L. A. Trant M. Weiman M. Knoll M. Federman N. C. (2019). A mobile-based mindfulness and social support program for adolescents and young adults with sarcoma: Development and pilot testing. JMIR mHealth and uHealth, 7(3), e10921. 10.2196/10921 30882352 PMC6441858

[bibr7-19375867211067542] Duchemin A. M. Steinberg B. A. Marks D. R. Vanover K. Klatt M. (2015). A small randomized pilot study of a workplace mindfulness-based intervention for surgical intensive care unit personnel: Effects on salivary α-amylase levels. Journal of Occupational and Environmental Medicine, 57(4), 393–399. 10.1097/JOM.0000000000000371 25629803 PMC4624325

[bibr8-19375867211067542] Fenko A. Loock C. (2014). The influence of ambient scent and music on patients’ anxiety in a waiting room of a plastic surgeon. Health Environments Research & Design Journal, 7(3), 38–59. 10.1177/193758671400700304 24782235

[bibr9-19375867211067542] Goldbeck L. Ellerkamp T. (2012). A randomized controlled trial of multimodal music therapy for children with anxiety disorders. Journal of Music Therapy, 49(4), 395–413. 10.1093/jmt/49.4.395 23705344

[bibr10-19375867211067542] Gutiérrez E. O. F. Camarena V. A. T. (2015). Music therapy in generalized anxiety disorder. The Arts in Psychotherapy, 44, 19–24. 10.1016/j.aip.2015.02.003

[bibr11-19375867211067542] Hargreaves D. J. Krause A. E. (2016). Music and consumer behaviour. In Hallam S. Cross I. Thaut M. (Eds.), Oxford handbook of music psychology (2nd ed.). Oxford University Press. 10.1093/oxfordhb/9780198722946.013.47

[bibr12-19375867211067542] Höller Y. Thomschewski A. Schmid E. V. Höller P. Crone J. S. Trinka E. (2012). Individual brain-frequency responses to self-selected music. International Journal of Psychophysiology, 86(3), 206–213. 10.1016/j.ijpsycho.2012.09.005 23000014

[bibr13-19375867211067542] Holm L. Fitzmaurice L. (2008). Emergency department waiting room stress: Can music or aromatherapy improve anxiety scores? Pediatric Emergency Care, 24(12), 836–838. 10.1097/PEC.0b013e31818ea04c 19050663

[bibr14-19375867211067542] Huang R. Shih Y. (2011). Effect of background music on concentration of workers. Work, 38(4), 383–387. 10.3233/wor-2011-1141 21508527

[bibr15-19375867211067542] Hyde R. Bryden F. Asbury A. J. (1998). How would patients prefer to spend the waiting time before their operations? Anaesthesia, 53, 192–195. 10.1046/j.1365-2044.1998.00268.x 9534647

[bibr16-19375867211067542] Jones M. Brittain D. (2009). Music in the waiting room. The British Journal of General Practice, 59(565), 613–614. 10.3399/bjgp09x453864 PMC2714788 22751239

[bibr17-19375867211067542] Kipnis G. Tabak N. Koton S. (2016). Background music playback in the preoperative setting: Does it reduce the level of preoperative anxiety among candidates for elective surgery? Journal of PeriAnesthesia Nursing, 31(3), 209–216. 10.1016/j.jopan.2014.05.015 27235957

[bibr18-19375867211067542] Lee D. Henderson A. Shum D. (2004). The effect of music on preprocedure anxiety in Hong Kong Chinese day patients. Journal of Clinical Nursing, 13(3), 297–303. 10.1046/j.1365-2702.2003.00888.x 15009332

[bibr19-19375867211067542] Lee K. C. Chao Y. H. Yiin J. J. Hsieh H. Y. Dai W. J. Chao Y. F. (2012). Evidence that music listening reduces preoperative patients’ anxiety. Biological Research for Nursing, 14, 78–84. 10.1177/1099800410396704 21278165

[bibr20-19375867211067542] Leubner D. Hinterberger T. (2017). Reviewing the effectiveness of music interventions in treating depression. Frontiers in Psychology, 8, 1109. 10.3389/fpsyg.2017.01109 28736539 PMC5500733

[bibr21-19375867211067542] Liu H. Gao X. Hou Y. (2019). Effects of mindfulness-based stress reduction combined with music therapy on pain, anxiety, and sleep quality in patients with osteosarcoma. Revista Brasileira de Psiquiatria, 41(6), 540–545. 10.1590/1516-4446-2018-0346 31116262 PMC6899366

[bibr22-19375867211067542] Panda A. Garg I. Shah M. (2015). Children’s preferences concerning ambiance of dental waiting rooms. European Archives of Paediatric Dentistry, 16(1), 27–33. 10.1007/s40368-014-0142-z 25249336

[bibr23-19375867211067542] Pelletier C. L. (2004). The effect of music on decreasing arousal due to stress: A meta-analysis. Journal of Music Therapy, 41(3), 192–214. 10.1093/jmt/41.3.192 15327345

[bibr24-19375867211067542] Ramdinmawii E. Mittal V. K . (2017). *The effect of music on the human mind: A study using brainwaves and binaural beats* [Conference session]. 2017 2nd International conference on telecommunication and networks (TEL-NET), August 10–11, IEEE, Noida, India.

[bibr25-19375867211067542] Ribeiro M. Alcântara-Silva T. Oliveira J. Paula T. C. Dutra J. Pedrino G. R. Simões K. Sousa R. B. Rebelo A. (2018). Music therapy intervention in cardiac autonomic modulation, anxiety, and depression in mothers of preterms: Randomized controlled trial. BMC Psychology, 6(1), 57. 10.1186/s40359-018-0271-y 30545420 PMC6292022

[bibr26-19375867211067542] Routhieaux R. L. Tansik D. A. (1997). The benefits of music in hospital waiting rooms. The Health Care Supervisor, 16(2), 31–40. 10174442

[bibr27-19375867211067542] Schäfer T. Sedlmeier P. Städtler C. Huron D. (2013). The psychological functions of music listening. Frontiers in Psychology, 4, 511. 10.3389/fpsyg.2013.00511 23964257 PMC3741536

[bibr28-19375867211067542] Shakir A. Chattopadhyay A. Paek L. S. McGoldrick R. B. Chetta M. D. Hui K. Lee G. K. (2018). The effects of music on microsurgical technique and performance: A motion analysis study. Annals of Plastic Surgery, 78(5 Suppl 4), S243–S247. 10.1097/SAP.0000000000001047 28399026

[bibr29-19375867211067542] Shallcross A. J. Willroth E. C. Fisher A. Dimidjian S. Gross J. J. Visvanathan P. D. Mauss I. B. (2018). Relapse/recurrence prevention in major depressive disorder: 26-Month follow-up of mindfulness-based cognitive therapy versus an active control. Behavior Therapy, 49(5), 836–849. 10.1016/j.beth.2018.02.001 30146148 PMC6112178

[bibr30-19375867211067542] Shih Y. N. Huang R. H. Chiang H. Y. (2012). Background music: Effects on attention performance. Work, 42(4), 573–578. 10.3233/WOR-2012-1410 22523045

[bibr31-19375867211067542] Silverman M. J. Christenson G. A. Golden D. Chaput-McGovern J. (2012). Effects of live music on satisfaction of students waiting for treatment in a university health clinic. Music Therapy Perspectives, 30(1), 43–48. 10.1093/mtp/30.1.43

[bibr32-19375867211067542] van Emmerik A. Berings F. Lancee J. (2018). Efficacy of a mindfulness-based mobile application: A randomized waiting-list controlled trial. Mindfulness, 9(1), 187–198. 10.1007/s12671-017-0761-7 29387266 PMC5770479

[bibr33-19375867211067542] Ventura T. Gomes M. C. Carreira T. (2012). Cortisol and anxiety response to a relaxing intervention on pregnant women awaiting amniocentesis. Psychoneuroendocrinology, 37(1), 148–156. 10.1016/j.psyneuen.2011.05.016 21705148

[bibr34-19375867211067542] Verheecke G. Troch E. (1980). Music while you wait. Patient acceptance of music in the preanesthetic period. Acta Anaesthesiologica Belgica, 31(1), 61–67. 7457045

[bibr35-19375867211067542] Waldon E. G. Thom J. C. (2015). Recorded music in the mental health waiting room: A music medicine investigation. The Arts in Psychotherapy, 46, 17–23. 10.1016/j.aip.2015.07.006

[bibr36-19375867211067542] Wetherell J. L. (1998). Treatment of anxiety in older adults. Psychotherapy: Theory, Research, Practice, Training, 35(4), 444–458. 10.1037/h0087745

